# Cost effectiveness of HIV and sexual reproductive health interventions targeting sex workers: a systematic review

**DOI:** 10.1186/s12962-018-0165-0

**Published:** 2018-12-04

**Authors:** Giulia Rinaldi, Aliasghar A. Kiadaliri, Hassan Haghparast-Bidgoli

**Affiliations:** 1 St. George’s University of London, London, UK; 2 Clinical Epidemiology Unit, Department of Clinical Sciences, Orthopaedics, Faculty of Medicine, Lund University, Lund, Sweden; 3 Institute for Global Health, University College London, 30 Guilford Street, London, WC1N 1EH UK

**Keywords:** Cost-effectiveness, HIV, Sexual reproductive health, CHEERS, DALY, Low and middle income, Sex workers

## Abstract

**Background:**

Sex workers have high incidences of HIV and other sexually transmitted diseases. Although, interventions targeting sex workers have shown to be effective, evidence on which strategies are most cost-effective is limited. This study aims to systematically review evidence on the cost-effectiveness of sexual health interventions for sex workers on a global level. It also evaluates the quality of available evidence and summarizes the drivers of cost effectiveness.

**Methods:**

A search of published articles until May 2018 was conducted. A search strategy consisted of key words, MeSH terms and other free text terms related to economic evaluation, sex workers and sexual and reproductive health (SRH) was developed to conduct literature search on Medline, Web of Science, Econlit and the NHS Economic Evaluation Database. The quality of reporting the evidence was evaluated using the CHEERS checklist and drivers of cost-effectiveness were reported.

**Results:**

Overall, 19 studies met the inclusion criteria. The majority of the studies were based in middle-income countries and only three in low-income settings. Most of the studies were conducted in Asia and only a handful in Sub-Saharan Africa and Latin America. The reviewed studies mainly evaluated the integrated interventions, i.e. interventions consisted a combination of biomedical, structural or behavioural components. All interventions, except for one, were highly cost-effective. The reporting quality of the evidence was relatively good. The strongest drivers of cost-effectiveness, reported in the studies, were HIV prevalence, number of partners per sex worker and commodity costs. Furthermore, interventions integrated into existing health programs were shown to be most cost-effective.

**Conclusion:**

This review found that there is limited economic evidence on HIV and SRH interventions targeting sex workers. The available evidence indicates that the majority of the HIV and SRH interventions targeting sex workers are highly cost-effective, however, more effort should be devoted to improving the quality of conducting and reporting cost-effectiveness evidence for these interventions to make them usable in policy making. This review identified potential factors that affect the cost-effectiveness and can provide useful information for policy makers when designing and implementing such interventions.

**Electronic supplementary material:**

The online version of this article (10.1186/s12962-018-0165-0) contains supplementary material, which is available to authorized users.

## Introduction

In past 2 decades, international agencies, in particular the United Nations and the Global Fund, have intensified their efforts on prevention of infectious diseases, especially HIV, through improving access to preventive and curative care [1]. The reasons for this are both humanitarian and financial, with sexually transmitted infections (STIs) known to hinder economic growth in low and middle income countries (LMICs), particularly in Sub-Sharan Africa, due to increased morbidity and mortality, as a result reducing labor supply and productivity [2]. In high-income, such as the Unites States, annual direct medical costs of sexually transmitted diseases (STDs) are estimated to be between USD 14 and USD 23 billion [3]. In past few decades, many strategies have been developed to control spread of HIV and STDs which include different forms of behavioural change and communication; condom promotion and distribution; voluntary counselling and testing; harm reduction strategies among drug users; STI prevention and treatment, antiretroviral/therapy (ART), mother-to-child transmission prevention interventions, pre-exposure prophylaxis (PrEP) and many other interventions. Evidence has shown that an appropriate mix of these interventions can lead to significant reductions in the prevalence of HIV and STDs [4–6].

Focusing on target groups with the highest rates of HIV, such as sex workers, has shown to have large impacts on reducing transmission to the whole population, and therefore, is likely to be highly cost-effective [7]. Sex workers, who defined as a population who exchange sex for money, goods or favours, are at a higher risk of adverse sexual health outcomes both due to their nature of work, their extraordinary social and economic vulnerability, and the high levels of stigma and violence attached to their work [8, 9]. Moreover, marginalised populations such as sex workers face many barriers accessing Sexual Reproductive Health (SRH) services due to reasons such as criminalisation of sex work, stigmatization and discrimination experienced at health facilities, increasing their vulnerability and obstructing their right to access health services [9–12]. Furthermore, sex workers lack of contact with SRH services is also influenced socio-demographics and the low levels of knowledge about the value of SRH interventions; for example, up to 26% of sex workers in Nigerian brothels were unaware of methods to protect against STIs [10, 13]. Interventions targeting barriers in access to SRH services amongst sex workers have shown to be very effective in reducing prevalence of HIV and STIs [14].

Several authors [14–16] have systematically reviewed the evidence for the cost-effectiveness of SRH interventions, especially HIV prevention strategies, aimed at the general population and concluded that there is limited economic evidence for some of the interventions as no economic analysis has been performed about these interventions, but for those that there was evidence many of them were cost-effective. However, evidence on what SRH interventions are most cost-effective amongst sex workers, the drivers of cost-effectiveness and the impact on the general population have rarely been evaluated [17].

The aim of this study was to systematically review the published evidence on cost-effectiveness of SRH interventions targeting sex workers. This review also aimed to assess the quality of the evidence and to identify the main drivers of the cost-effectiveness results amongst these interventions.

## Methods

### Search strategy

A literature search was conducted on Medline, Web of Science, Econlit and the NHS Economic Evaluation Database. The keywords used were divided into three groups: “cost-effectiveness”, “sexual reproductive health intervention” and “sex worker”. The full keywords used are shown in the Additional file 1, these were used in combination with each other.

### Inclusion and exclusion criteria

We included studies that were published from January 1995 to May 2018 in English, with a full economic evaluation, i.e. studies performed comparative analysis of costs and outcomes of at least two interventions. Full economic evaluation analyses include cost minimization analysis (CMA), cost-effectiveness analysis (CEA), cost-utility analysis (CUA) or cost–benefit analysis (CBA) [18]. We included the studies that specifically focused on sex workers or presented separate cost-effectiveness results for sex workers. Interventions included were all types of SRH interventions including preventative, curative or education or information provision that were targeted towards improving the SRH of sex workers. The full exclusion and inclusion criteria are presented in detail in Additional file 2.

### Data extraction and quality assessment

Relevant papers were selected in two steps: in first step, titles and abstracts of papers were screened and in second step entire papers were screened according to the inclusion criteria. The papers were screened independently by GR and HHB and disagreements were resolved by discussion.

A detailed analysis of the selected papers was carried out using a tool that was adapted from the existing guidelines and other review articles of economic evaluations [18–20]. Using this tool, general information and economic features of the selected papers were extracted. The summary table of these features can be seen in Table 1 and full details are presented in Additional file 3.

**Table 1:** Summary of economic features of the studies

Feature	N	%
Type of economic evaluation		
CEA	8	42
CUA	11	58
CMA	0	0
CBA	0	0
Study design		
Randomised clinical trial (RCT)	0	0
Observational	4	21
Modelling	15	79
Perspective evaluated		
Government/public sector	4	21
Provider	8	42
Program provider	2	11
Not specified	5	26
Time horizon		
≤ 1 year	4	21
1–10 years	4	21
Over 10 years/lifetime	5	26
Not specified	6	32
Type of outcome		
QALY/DALY	11	58
Infection averted	12	63
Infections cured	3	16
Level of care and intervention type		
Behavior change	1	5
Biomedical interventions	4	21
Structural interventions	1	5
Mixed	13	68
Type of data used		
Primary data	2	11
Secondary data	15	79
Mixed	2	11
Type of sensitivity analysis		
One-way/univariate	12	63
Multi-way/multivariate	8	42
Probabilistic analysis	6	32
Not performed/specified	2	11

*CUA* cost-utility analysis, *CEA* cost-effectiveness analysis, *CMA* cost-minimisation analysis, *CBA* cost-benefit analysis, *QALY* quality-adjusted life year, *DALY* disability-adjusted life year

The interventions included in this review were classified into four main categories: behavioral, biomedical, structural and mixed interventions. The behavioral or behavior change interventions aim to reduce the risk of HIV/STD infection through modification of sexual and substance use-related behaviors [21, 22]. These interventions seek to delay onset of sexual intercourse, decrease the number of sexual partners, reduce incidence of unprotected sex, reduce sharing of needles and syringes, and reduce or eliminate substance use. These interventions generally focus on counseling individuals, couples, peer groups or networks, institutions, and entire communities through different means such as peer groups, workshops, social networks, social marketing or mass media [21, 22]. Biomedical interventions use chemical and physical technology to prevent infection or decrease infectiousness, through targeting biological and physiological processes that are responsible for HIV acquisition and transmission. These include interventions such as male and female condoms, treatment of STIs, pre- or post-exposure prophylaxis, ART, male circumcision, microbicides, and vaccines [21, 23]. Structural interventions, also known as social, environmental, ecological, or upstream interventions, aim to change the underlying determinants of risk, vulnerability or disease. These interventions, which are varied in nature, include legal changes, microfinance, vouchers, women empowerment, income-generating activities, etc. [21, 24]. Mixed or integrated interventions are those interventions that consisted a combination of biomedical, structural, or behavioral strategies.

The quality of reporting the economic evaluation evidence was assessed using the Consolidated Health Economic Evaluation Reporting Standards (CHEERS) checklist [25]. Twenty-four items of the checklist were scored using ‘yes’ (met the criteria in full), ‘partially met’, ‘no’ (not met), and ‘not applicable’. GR and HHB independently assessed the papers using the checklist and any discrepancies were resolved by discussion.

### Interpreting cost-effectiveness of the interventions

The cost-effectiveness of the interventions was judged using the WHO recommendation on cost-effectiveness threshold stating that an intervention is highly cost-effective if cost-effectiveness ratio (cost per DALY averted) is less than the GDP per capita, is cost-effective if it is between one and three times the GDP per capita and it is not cost-effective if it is more than triple the GDP per capita [26]. In addition to the WHO recommendation, the cost-effectiveness results were judged using alternative thresholds, stating that an intervention, in low and middle income countries, is cost-effective if the cost-effectiveness ratio is < 50% of GDP per capita [27]. The outcomes reported in the studies, mainly in terms of cost per disability-adjusted-life-years (DALYs) averted, cost per quality-adjusted-life-years (QALYs) gained or cost per HIV infection (or STI) averted, were used in a cross-study comparison. For the studies that only reported cost per infection averted, the cost per infection averted was converted to cost per DALYs averted, using a conversion factor used by previous studies [16, 28, 29] (i.e. it was assumed that cost per DALY averted was equal to cost per infection averted divided by 20). To facilitate the cross-study comparison, all cost-effectiveness ratios were inflated to 2016 values using the consumer price indices for the country where the study conducted and then converted to 2016 international dollar (INT$) using the 2016 Purchasing power parity (PPP) conversion factor for each country [30].

## Results

### Search results

**Fig. 1:**
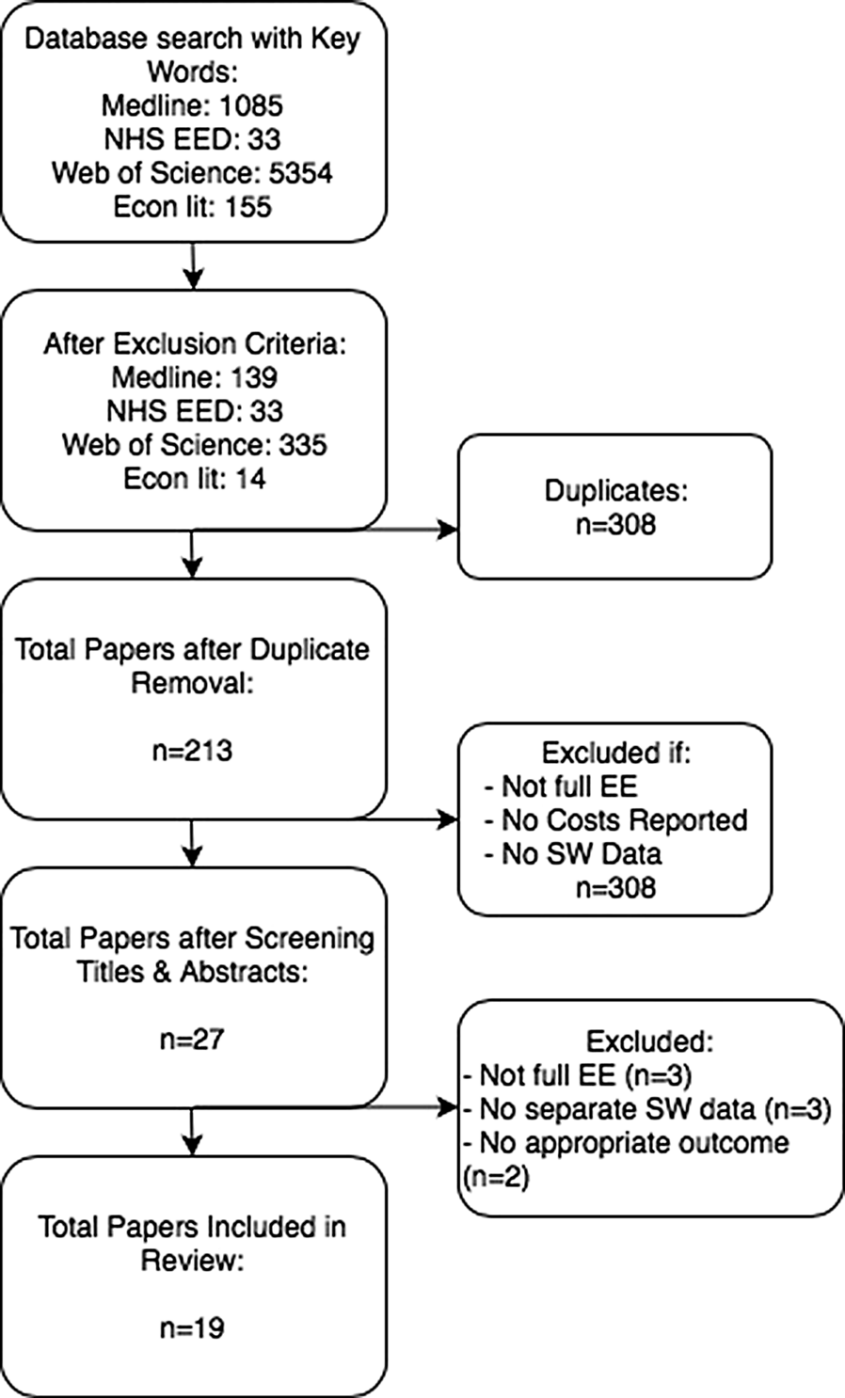
Literature search flowchart

The literature search identified a total of 6627 papers (Fig. 1). Initial title and abstract scanning excluded partial economic evaluations (i.e. cost description, cost of illness studies) and identified studies that provided data for sex workers. Through this process 27 potential papers were identified and analyzed and eight were excluded either due to lack of cost data, appropriate outcome or separate sex worker results. The reference lists for the remaining 19 studies [31–49] were scanned for other possible studies.

### General and economic features of the papers

The majority (74%, N = 14) of 19 included studies were based in middle-income countries with the exception of three low-income and two high-income settings. Most of the studies were conducted in Asia (48%, N = 10) following by sub-Saharan Africa (25%, N = 5).

A majority of the studies (58%, N = 11) were CUA, using DALYs (n = 8) or quality-adjusted life years (QALYs, n = 3) as the outcome measures. The remaining were CEA, using cases of infection prevented as the outcome. Overall, 15 out of the 19 (79%) studies employed modeling, four were observational studies, and there was no randomized control trial design. The perspectives were mostly provider (42%, N = 8) followed by government/public sector perspective (21%, N = 4), and five studies didn’t specify their perspective.

Six studies (32%) did not specify time horizon, 5 (26%) studies had a time horizon of over 10 years, 4 (21%) studies had a time horizon of 1–10 years and 4 (21%) had a time horizon of < 1 year. There was one behavioral intervention, which consisted of voluntary counseling and testing, 4 (21%) biomedical interventions, including STI test and treatment, HIV vaccination, introducing female condom, and 1 (5%) structural interventions, including provision of vouchers for sex workers. Furthermore, there were 13 (68%) mixed interventions, which consisted of either all three types of interventions or a combination of two. Majority of (n = 8, 62%) of mixed intervention included a combination of behavioral and biomedical strategies. Detailed features of the selected studies are reported in Additional file 3 and summarized in Table 1.

### Quality of reporting the evidence

**Fig. 2:**
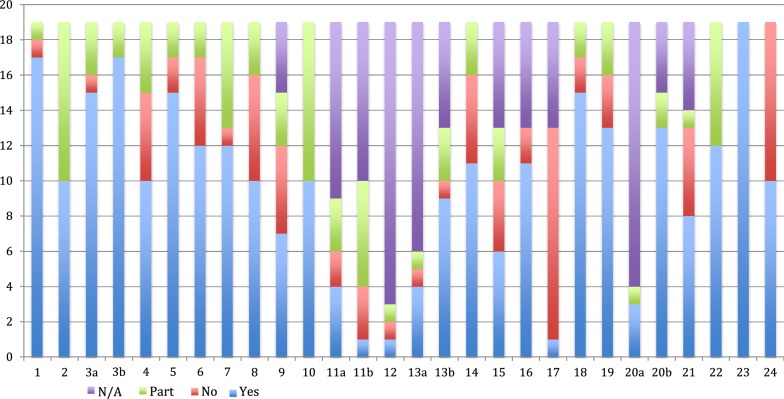
Quality of reporting economic evaluation of HIV and SRH interventions for sex workers per items of the CHEERS checklist. *SRH* sexual and reproductive health, *CHEERS* Consolidated Health Economic Evaluation Reporting Standards, *N*/*A* not applicable, *No* not reported, *Yes* reported

The findings of the assessment of reporting quality using the CHEERS checklist are presented in Additional file 4 and summarized in Table 2 and Fig. 2. The findings showed that on average, the compliance with each item on the CHEERS checklist was 62% (ranging from 8 to 100%). The most frequently not reported items were item 9 ‘discount rate’ (47% compliant), item 11b ‘measurement of effectiveness in synthesis-based estimates’ (10% compliant) and item 12 ‘measurement and valuation of preference based outcome’ (33% compliant). Furthermore, item 15 ‘choice of model’ (46% compliant) and item 17 ‘describing analytical models’ (8% compliant) were major areas of weakness for the included studies.

**Table 2:** Number of studies fulfilling each CHEERS checklist items

Item	Item no.	Yes	No	Partially	NA
Title and abstract					
Title	1	17	1	1	0
Abstract	2	10	0	9	0
Introduction					
Background and objectives	3a	15	1	3	0
	3b	17	0	2	0
Methods					
Target population and subgroups	4	10	5	4	0
Setting and location	5	15	2	2	0
Study perspective	6	12	5	2	0
Comparators	7	12	1	6	0
Time horizon	8	10	6	3	0
Discount rate	9	7	5	3	4
Choice of health outcomes	10	10	0	9	0
Measurement of effectiveness	11a	4	2	3	10
	11b	1	3	6	9
Measurement and valuation of preference based outcomes	12	1	1	1	16
Estimating resources and costs	13a	4	1	1	13
	13b	9	1	3	6
Currency, price date, and conversion	14	11	5	3	0
Choice of model	15	6	4	3	6
Assumptions	16	11	2	0	6
Analytical methods	17	1	12	0	6
Results					
Study parameters	18	15	2	2	0
Incremental costs and outcomes	19	13	3	3	0
Characterising uncertainty	20a	3	0	1	15
	20b	13	0	2	4
Characterising heterogeneity	21	8	5	1	5
Discussion					
Study findings, limitations, generalisability, and current knowledge	22	12	0	7	0
Other					
Source of funding	23	19	0	0	0
Conflicts of interest	24	10	9	0	0

### Cost-effectiveness by intervention type, income level and geographical region

Table 3 presents the cost-effectiveness results of the selected studies, classified based on the intervention type and ranked according to incremental cost-effectiveness ratios (ICERs). Except for one study (that evaluated regular mandatory STIs testing for sex workers in Victoria, Australia [48] which was not cost-effective), all other interventions were highly cost-effective, according to the WHO threshold [26] as well as Woods et al.’s [27] alternative threshold of ICER less than 50% of GDP per capita.

**Table 3:** Cost effectiveness results of the reviewed studies based on the type of the intervention, ranked by cost-effectiveness ratio

Paper	Intervention components	GDP/capita of the country—2016 INT$	Original ICER reported	Cost/DALY averted—2016 INT$	Other outcomes—2016 INT$
**Behavioral interventions**					
Tromp et al. [44]	VCT for HIV and condom use	11,632	248 (US$ 2008)/HIV infection averted and 9.2 (US$ 2008)/DALY averted	33	889/HIV infection averted
**Biomedical interventions**					
Carrara et al. [34]	STI services (diagnosis and treatment)	3744	127 (US$ 2002)/STI cured	33^a^	658/STI cured
Marseille et al. [40]	Female condom	13,248	509 (US$ 2000)/HIV infection averted	62^a^	1232/HIV infection averted
Leelahavarong et al. [39]	HIV vaccination	16,946	2840 (Thai Bhat 2009)/QALY gained	264	NA
Wilson et al. [48]	STI testing for HIV, chlamydia and gonorrhea testing every 12 weeks	46,790	10,000,000 (AUS$ 2007)/QALY gained	10,197,390	4078,956/HIV infection averted
**Structural interventions**					
Borghi et al. [32]	Vouchers for free SH consul and STI treat	5550	103 (US$ 1999)/STI cured	18^a^	364/STI cured
**Mixed interventions**					
Hutton and Wyss [38]	Mass media, social marketing of condoms, peer group education, treatment of STIs, prevention of mother-to-child-transmission, blood safety and voluntary testing	1996	16 (US$ 2012)/HIV infection averted	4^a^	77/HIV infection averted
Vassal et al. [45]	Community mobilisation and empowerment (i.e. community involvement in programme management and services, violence reduction, and addressing legal policies and police practices) was added to Avahan’s existing interventions. (behavior change, condom distribution, STI treatment, etc.)	6583	13.48 (US$ 2011)/DALY averted and 228 (US$ 2011)/HIV infection averted	51	869/Infection averted in Bellary
			14.12 (US$ 2011)/DALY averted and 234 (US$ 2011)/HIV infection averted	54	892/infection averted in Belgaum
Burgos et al. [33]	Mujer Segura: 35 min behavioral skills session to improve condom negotiation provided once-only or annually, with or without HAART	17,877	183 (US$ 2009)/DALY averted	368	4779/HIV infection averted
Sweat et al. [43]	Community mobilisation, promotional media, and interpersonal communication (and a policy regulatory intervention in Puerto Playa)	15,2355	1186 (US$ 2006)/DALY averted	3017	Santo Domingo: 71,747/HIV infection averted
			457 (US$ 2006)/DALY averted	1162	Puerto Playa: 27,612/HIV infection averted
Vickerman et al. [46]	Rapid test for HIV, chlamydia and gonorrhea plus condom promotion	2172	151 (US$ 2004)/HIV infection averted	25^a^	503/HIV infection averted
Fung et al. [36]	Behavioral, STI treatment, peer-education, Condom distribution	6583	98 (US$ 2007)/HIV infection averted	31^a^	626/HIV infection averted
Prinja et al. [42]	Peer-led counseling, condom promotion, quarterly health check-up, STI treatment, and HAART	6583	106 (US$ 2009)/HIV infection averted and 11 (US$ 2009)/DALY averted	53	509/HIV infection averted
Aldridge et al. [31]	Peer counselling and STI treatment including condom distribution	13,044	55 (US$ 2008)/DALY averted	130	NA
Panovska-Griffiths et al. [41]	Peer mediated communications strategies, social marketing of condoms and management of STI; and enabling environment for the adoption of safer sex practices	6583	720 (US$ 2007)/HIV infection averted	173^a^	3463/HIV infection averted
Dandona et al. [35]	Behaviour change communication, STIs care, condom promotion, and creating an enabling environment	6583	984 (US$ 2005)/HIV infection averted	227	5868/HIV infection averted
Vickerman et al. [47]	Mobile clinic with syndromic screening and treatment of STIs, condom distribution, health education with and without periodic presumptive treatment in hotels	13,248	78 (US$ 2001)/DALY averted and 2093 (US$ 2001)/HIV infection averted	301	8063/HIV infection averted
You et al. [49]	STI testing, education, condom distribution, negotiation skills, video presentation, role plays, and peer group discussions	58,651	10,315 (US$ 2002)/gonorrhea or chlamydia infection averted	959^a^	19,175/gonorrhea or chlamydia infection averted

Hogan et al. study [37] was not inserted into the table as it was provided modeled estimates for two WHO regions and it was difficult to convert these estimated to 2016 international dollar

*GDP* gross domestic product, *INT* international dollar, *VCT* voluntary counselling and testing, *PPTCT* prevention of parent to child transmission, *HAART*  highly-active anti-retroviral therapy

^a^Estimated ratio. For studies that cost per DALY averted was not available, it was assumed that cost per DALY averted was equal to cost per infection averted divided by 20 [16, 28, 29]

There was only one behavioral intervention (included promoting voluntary counseling and testing and condom use components), implemented in West-Java [44] which costs INT$33 per DALY averted (or INT$889 per HIV infection averted).

Four studies (21%) evaluated cost-effectiveness of biomedical interventions. In this category, a facility-based STI case management study in a province in Colombia by Carrara et al. [34] showed that the cost per STI treated was INT$658. Contrastingly, Wilson et al. [48] demonstrated that the current scheme in Australia for sex workers’ STI checkups costs the government INT$4,078,956 per HIV infection averted, which, is not cost effective.

Only one study (5%) implemented structural interventions, which was a competitive voucher scheme in Managua, Nicaragua to increase STI testing and treatment uptake in high-risk groups (such as female sex workers and their clients, transvestites etc.) [32]. ICER for this intervention was INT$364 per STI cured.

The ICER among the remaining 13 (48%) mixed interventions ranged from INT$51 per DALY averted (or INT$869 per HIV infection averted) in a district in India, for adding a structural component (i.e. community empowerment and involvement in programme management and services, violence reduction, and addressing legal policies and police practices) to existing behavioral and biomedical strategies [45], to INT$3017 per DALY averted (INT$71,747 per HIV infection averted) in Santo Domingo district in Dominican Republic, for an intervention package consisted of community mobilization activities (such as educational workshops and materials), promotional media (such as posters and stickers to reinforce the message that the sex establishments were ‘‘100% condom’’ settings), and interpersonal communication (such as informational booths, and interactive theater presentations, training sex workers as health educators/counselors and peer facilitators in STI clinics) [43].

**Fig. 3:**
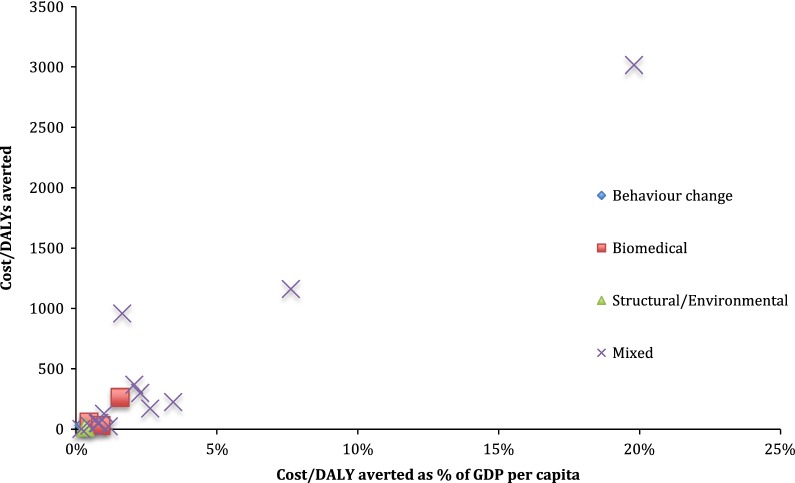
Cost per DALY averted vs. cost per DALY averted as % of GDP per capita, by intervention type. For studies that cost per DALY averted was not available, it was assumed that cost per DALY averted was equal to cost per infection averted divided by 20 [16, 28, 29]. Two studies [39, 48] used cost per QALY gained. Wilson et al. [48] study was outlier (with ICER of over INT$10 million) and was not included in the graph. Hogan et al. [37] study was not included as it was provided modeled estimates for two WHO regions and it was difficult to convert these estimated to 2016 international dollar. Separate ICER was included for the studies [43, 45] that reported ICER separately for the cities/districts the intervention implemented

With the exception of the Wilson et al. study [48] in Australia, in all reviewed settings, the ICERs of all other interventions is < 20% of the GDP per capita, proving to be highly cost-effective interventions (Fig. 3). The ICERs of biomedical interventions were consistently less than 2% of GDP per capita. Similarly, ICERs of the mixed interventions are on average 3% of GDP per capita (ranging from 0.2 to 20%).

**Fig. 4:**
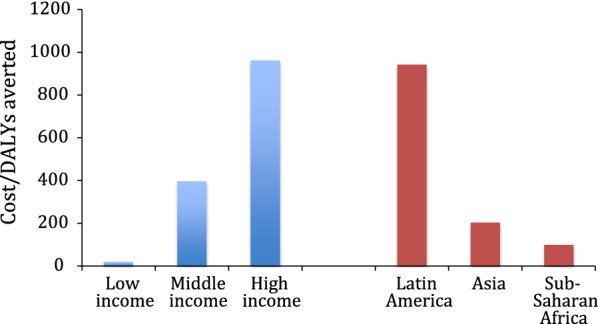
Comparing average cost-effectiveness ratios of the interventions based on the income level and geographical region. For studies that cost per DALY averted was not available, it was assumed that cost per DALY averted was equal to cost per infection averted divided by 20 [16, 28, 29]. Two studies [39, 48] used cost per QALY gained. Wilson et al. [48] study was outlier (with ICER of over INT$10 million) and was not included in the graph. Hogan et al. [37] study was not included as it was provided modeled estimates for two WHO regions and it was difficult to convert these estimated to 2016 international dollar. Separate ICER was included for the studies [43, 45] that reported ICER separately for the cities/districts the intervention implemented

ICER of SRH interventions in low-income countries ranged from INT$4 to INT$33 per DALYs averted, compared to INT$18–INT$3017 in middle income countries (Fig. 4). ICERs also varied in different geographical areas, the lowest in sub-Saharan Africa (on average, INT$98, ranging from INT$4 to INT$310) and the highest in Latin America (on average, INT$939, ranging from INT$18 to INT$3017) (Fig. 4).

### Cost-effectiveness by intervention structure and platform of delivery

**Fig. 5:**
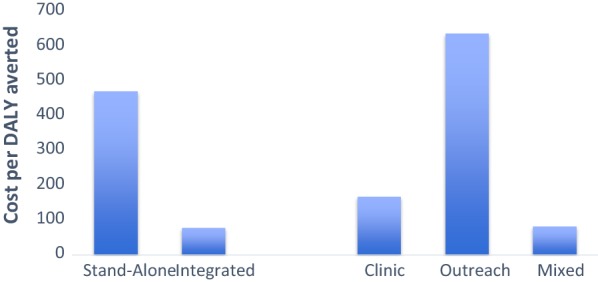
Comparing average cost-effectiveness ratios of the interventions based on the structure (stand-alone vs. integrated) and platform of delivery (clinic-based vs. outreach-based or mixed). For studies that cost per DALY averted was not available, it was assumed that cost per DALY averted was equal to cost per infection averted divided by 20 [16, 28, 29]. Two studies [39, 48] used cost per QALY gained. Wilson et al. [48] study was outlier (with ICER of over INT$10 million) and was not included in the graph. Hogan et al. [37] study was not included as it was provided modeled estimates for two WHO regions and it was difficult to convert these estimated to 2016 international dollar. Separate ICER was included for the studies [43, 45] that reported ICER separately for the cities/districts the intervention implemented

Majority (n = 13) of the interventions evaluated in this review were stand-alone interventions and only five interventions were integrated into the existing programs. On average, ICER for the integrated interventions was INT$79 (ranging from INT$18 to INT$264), while ICER for stand-alone intervention was INT$469 (ranging from INT$4 to INT$3017) (Fig. 5).

Majority of the interventions were either delivered as outreach (n = 7) or at the clinic or health care facility (n = 6) and only five interventions were delivered using both platforms. Examining the ICER by platform of delivery indicated that on average mixed platform interventions had lower ICER, with INT$83 (ranging from INT$25 to INT$173), compared to clinic-based (with average ICER of INT$169, ranging from INT$18 to INT$301) and outreach (with average ICER of INT$634, ranging from INT$4 to INT$3017) interventions (Fig. 5).

### Drivers of cost effectiveness

The drivers of cost-effectiveness were evaluated based upon the results from one-way/univariate sensitivity analyses. Most (89%, N = 17) studies conducted sensitivity analyses including one-way/univariate (63%, n = 12), multivariate (42%, N = 8) and probabilistic (32%, N =  6 analyses. The remaining 2 (11%) studies did not perform or specify any sensitivity analysis. Additional file 5 summarizes the results of the sensitivity analyses conducted in each study and the main drivers of cost effectiveness reported by the authors.

Studies showed [33, 40, 43] that the cost-effectiveness of HIV and SRH interventions for sex workers was most sensitive to HIV incidence and prevalence amongst sex workers. For example, Burgos et al. [33] showed that when incidence of HIV amongst sex workers increased to 4% the cost per QALY decreased to 122$ per QALY gained whilst, when it fell to 0.3% the cost per QALY substantially increased (US$1202 per QALY gained). The number of partners per sex worker was also a recurring factor causing significant changes in cost-effectiveness results. Marseille et al. [40] reported that the numbers of partners per sex worker (or female condom user in this context) altered the cost-effectiveness results, indicating that more clients per year resulted in the female condom to be more cost saving. You et al. [49] demonstrated their results were sensitive to the number of clients per day and contact rate between female sex workers with their regular partners.

Commodity costs were another determinant factor in the cost-effectiveness in some studies. One study [47] reported that the cost of rapid diagnostic test was the largest determinant of the ICER. Changes in personnel costs and laboratory test prices caused significant changes in the cost-effectiveness outcomes in Borghi et al. [32] study. Another driver of cost effectiveness results, which was mentioned in several studies, was the effectiveness of the intervention services provided such as the impact of voluntary counselling and testing (VCT) on condom use [44] or the actual effectiveness of female condoms in preventing STI transmissions [40].

Sweat et al. [43] found that cost-effectiveness of HIV and SRH interventions are sensitive to the choice of discount rate and marginally sensitive to STD prevalence in sex workers. However, Vassall et al. [45] stated that variations in discount rate did not increase the incremental cost per DALY averted above the cost-effective thresholds suggested by the WHO. In summary, the most important drivers to rendering a SRH intervention cost-effective amongst sex workers is the high incidence and prevalence of HIV and other STIs, the average number of partners each sex worker has per year, and lastly, the commodity costs of the intervention.

## Discussion

This is, to our best knowledge, the first study that has systematically reviewed the cost-effectiveness evidence of HIV and SRH interventions amongst sex workers, specifically focusing on identifying the drivers of cost-effectiveness. The findings of this study show that there is limited data on the cost-effectiveness of HIV and SRH interventions among sex workers, in particular, new developed interventions such as PrEP. The findings also strongly suggest that HIV and SRH interventions for sex workers are highly cost-effective as per international and national cost-effectiveness thresholds. The findings also suggest that quality of reporting evidence was relatively good but needs improvement in some areas. Moreover, the findings showed that the main drivers of cost-effectiveness of the interventions were HIV incidence and prevalence amongst sex workers, number of sexual partners of sex workers, and commodity costs.

The findings demonstrate that all HIV and SRH interventions targeted at sex workers were highly cost-effective except for one. This is consistent with previous reviews evaluating cost-effectiveness of HIV prevention strategies in the general population [16]. The cost-effectiveness of the interventions was partially attributed to economies of scale and synergies caused by bundling several interventions together under one program or adding the new interventions to the existing programs. Most studies evaluated a package of prevention or prevention and treatment interventions and around a third of the interventions integrated to the current health care system or programs on the ground (Additional file 3). For example, Dandona et al. [35], evaluated a program consist of behaviour change, STI care, condom promotion, and creating an enabling environment components, and Fung et al. [36] evaluated a strategy that includes STI treatment, peer education and condom distribution.

The findings also suggest that quality of reporting cost-effectiveness evidence is not ideal and needed to be improved in order to provide a useful source of information for decision makers. The specific areas for improvement identified by this review are justification for the choice of model and clear description of underpinned assumptions, transparency in synthesizing the clinical effectiveness data, specifying the time horizon, and the perspective of evaluation. In addition, it is recommended that future studies use comprehensive measures such as DALY or QALY, as gold standard measurements, to allow comparability across broad range of diseases [18] and adopt a societal perspective (instead of narrower perspectives, such as payer or provider) in order to facilitate optimal resource allocation decisions [50, 51] and enhance the comparability across different sectors.

The main drivers of cost effectiveness reported in the reviewed papers were HIV incidence and prevalence amongst sex workers, numbers of partners per sex worker, and commodity costs. This is in line with the results of a previous systematic review which reported that cost-effectiveness of HIV prevention strategies was sensitive to the population targeted, the size of the intervention, and the unit costs [16]. Additionally, Cohen et al. [7] found that the drivers of cost effectiveness of HIV prevention interventions were HIV prevalence and cost per person reached.

As mentioned earlier, other potential factors that might affect the cost-effectiveness of the interventions, due to economies of scope, are bundling the interventions together under one program as well as embedding the new interventions to the existing programs (Figs. 3 and 5). For instance, the interventions that were integrated to the existing programs, on average, were more cost-effective than the stand-alone interventions (Fig. 5). A stand-alone intervention might result in greater costs as it does not have the capital, infrastructure and staffing support of an established service, therefore, is often thought to be less sustainable [52]. Moreover, this supports the idea that integrated programs can be more cost-effective due to the ability to originate from scaling-up existing programs to reach desirable populations such as sex workers [53]. Similarly, whether the intervention is an outreach or clinic/facility based or mixed intervention might affect the cost-effectiveness results. Mixed outreach and facility-based interventions, on average, were more cost-effective than pure outreach interventions (Fig. 5).

### Potential limitations

This review has several potential limitations that should be considered when interpreting its findings. Firstly, it is not possible to differentiate which intervention types are more cost-effective than others, since the studies reviewed did not take into account interactions between different types of interventions. Moreover, the cost-effectiveness of each intervention is not directly compared, so it is difficult to determine the optimal intervention. Further analyses, including modelling, are needed to comparatively assess the cost-effectiveness of each intervention in the same population to determine which intervention is most cost-effective. In addition, comparisons of the results between different studies and countries are problematic since the interventions evaluated are not homogenous, and cost-effectiveness results are influenced by a variety of factors such as epidemiological characteristics, coverage, unit prices or supply costs and the technical efficiency for implementation.

There are also few potential limitation in regards to the literature review process that need to be considered. This study only included published studies implying that the results might have skewed by publication bias. Furthermore, as in any review study, it is difficult to rule out selection bias or disagreement between the criteria of the reviewers. To minimize this bias, we used pre-defined inclusion criteria and discussion of disagreement between the investigators throughout the review process. Lastly, for cross study comparison, we used a conversion factor of 20 to convert cost per infection averted to cost per DALYs averted for the studies that cost per DALY averted was not available (n = 8). This conversion factor, has been widely used in the literature [16, 28, 29] and originated from the 1996 Global Burden of Disease [54] study when the HIV epidemic did not necessarily have the same characteristics as the present time. Research to create a contemporary conversion factor to convert non-DALY estimates to cost per DALY ratios is ongoing [55].

## Conclusion

The findings of this review show that there is limited economic evidence on HIV and SRH interventions targeting sex workers. The available evidence indicates that the majority of the interventions are highly cost-effective considering national and internationally recognized thresholds, however, more effort should be devoted to improving the quality of (conducting and) reporting cost-effectiveness evidence for these interventions to make them usable in policy making. This review identified potential factors that affect the cost-effectiveness and can provide useful information for policy makers when designing and implementing such interventions.

## Additional files

**Additional file 1.** Key search words.

**Additional file 2.** Inclusion and exclusion criteria.

**Additional file 3.** Detailed economic characteristics of the reviewed studies.

**Additional file 4.** Quality of reporting cost-effectiveness results for the interventions using CHEERS

**Additional file 5.** Sensitive analysis results and drivers of cost-effectiveness.

## Abbreviations

STI: sexually transmitted diseases
SRH: sexual and reproductive health
CMA: cost minimization analysis
CEA: cost-effectiveness analysis
CUA: cost-utility analysis
CBA: cost–benefit analysis
CHEERS: Consolidated Health Economic Evaluation Reporting Standards
DALYs: disability-adjusted life years
QALYs: quality-adjusted life years
ART: antiretroviral therapy
ICER: incremental cost-effectiveness ratio
PrEP: pre-exposure prophylaxis
