# Supplementary material for: Cost effectiveness of HIV and sexual reproductive health interventions targeting sex workers: a systematic review
Source: Cost Eff Resour Alloc. 2018 Dec 4;16:63. doi: 10.1186/s12962-018-0165-0 (PMC6278021; doi:10.1186/s12962-018-0165-0)
Supplement: Supplementary file 1 — Additional file 1. Key search words. [file 12962_2018_165_MOESM1_ESM.docx]

Additional file 1: Key Search Words

| **Cost-effective** | **Sexual Reproductive Health Interventions** | **Sex workers** |
| --- | --- | --- |
| Cost  Cost analysis  Economic evaluation  Cost saving  Cost Effectiv*  Cost-Effectiv*  Cost utility  Efficien*  Cost minimization  Cost consequence  Effectiv*  Cost benefit  Cost effective | Health intervention  Healthcare  SRH  Sexual health  Test*  HIV *  HIV/AIDS  Sexually transmitted disease*  STD*  Sexually transmitted infection*  STI*  Chlamydia  Condom*  Contracepti*  Family Planning  Screening  Reproductive tract infection*  RTI*  Voluntary counseling and testing  VCT*  Fertility | Prostitute*  Female sex worker*  Sex worker*  Male sex worker* |
