# Supplementary material for: Cost effectiveness of HIV and sexual reproductive health interventions targeting sex workers: a systematic review
Source: Cost Eff Resour Alloc. 2018 Dec 4;16:63. doi: 10.1186/s12962-018-0165-0 (PMC6278021; doi:10.1186/s12962-018-0165-0)
Supplement: Supplementary file 2 — Additional file 2. Inclusion and exclusion criteria. [file 12962_2018_165_MOESM2_ESM.docx]

Additional file 2: Inclusion & Exclusion Criteria

| **Inclusion Criteria** | **Exclusion Criteria** | **Rationale** |
| --- | --- | --- |
| English | Non-English | Readability |
| Published in peer reviewed journal | Non-Published | Quality |
| Year 1995 Onwards | Conference Papers | Generalizability |
| Original Studies | Not original studies | Avoids interpretation bias |
| Full economic evaluation study (i.e., cost minimization analysis [CMA], cost-effectiveness analysis [CEA], cost-utility analysis [CUA] or cost-benefit analysis [CBA]) (18). | Partial Economic Evaluations (i.e. cost-of-illness, cost description etc.) (18) | We are looking at cost effectiveness |
| Any intervention including preventative, curative or informative strategies. Could be structural, biomedical or behavioral interventions. |  |  |
