# Supplementary material for: Cost effectiveness of HIV and sexual reproductive health interventions targeting sex workers: a systematic review
Source: Cost Eff Resour Alloc. 2018 Dec 4;16:63. doi: 10.1186/s12962-018-0165-0 (PMC6278021; doi:10.1186/s12962-018-0165-0)
Supplement: Supplementary file 3 — Additional file 3. Detailed economic characteristics of the reviewed studies. [file 12962_2018_165_MOESM3_ESM.docx]

Additional file 3: Detailed economic characteristics of the reviewed studies

| **Paper** | **Type of EE** | **Study Design** | **Perspective** | **Time Horizon** | **Country** | **Outcome measures** | **Stand-alone or integrated** | **Clinic-based/outreach or mixed** | **Implementer** | **Type of sex worker** | **HIV/STI prevalence amongst sex workers** | **Sample Size** |
| --- | --- | --- | --- | --- | --- | --- | --- | --- | --- | --- | --- | --- |
| Aldridge (2009) | CUA | Modelling | Not Specified | Not Specified | Peru | DALYs averted | Stand-alone | Mixed | NGO | FSW | 1.8% | 27,274,000 |
| Borghi (2005) | CEA | Observational (Retrospective) | Provider | 1 year (Feb. 1999-2000) | Nicaragua | STI cured | Integrated | Clinic | Government | All | 2% | Not Specified |
| Burgos (2010) | CUA | Modelling | Government health care payer | Life Time | Mexico | QALY gained and HIV case averted | Stand-alone | Outreach | Government | FSW | 6% | 709 |
| Carrara (2005) | CEA | Observational (Retrospective) | Provider | 30 months (not specified exactly) | Cambodia | STI syndrome treated and cured. | Stand-alone | Clinic | NGO | All | 41% | 11330 of which 1012 were FSW |
| Dandona (2010) | CUA | Observational (Retrospective) | Not Specified | 1 year (2005-2006 Fiscal Year) | India | DALY averted & HIV infection averted | Stand-alone | Clinic | Government & NGO | FSW | 9.7% | 16 Programs |
| Fung (2007) | CEA | Modelling | Provider | 51 Months | India | HIV Infection averted | Stand-alone | Mixed | Government | FSW | 13% | 385 |
| Hogan (2003) | CUA | Modelling | Not Specified | Not Specified | Sub-Saharan Africa & SE Asia | DALYs averted/ Infection averted | - | - | - | FSW | - | Not Specified |
| Hutton (2013) | CEA | Modelling | Not Specified | Not Specified | Chad | Infection averted | Stand-alone | Outreach | Government | All | 15% | Depends on Source |
| Leelahavarong (2001) | CUA | Modelling | Government | Life Time (99 Years) | Thailand | QALY gained | Integrated | Clinic | Government | All | 20% | Not Specified |

Additional file 3- continued

| **Paper** | **Type of EE** | **Study Design** | **Perspective (as stated by authors)** | **Time Horizon** | **Country** | **Outcome measures** | **Stand-alone or integrated** | **Clinic-based/ outreach or mixed** | **Implementer** | **Type of sex worker** | **HIV/STI prevalence amongst sex workers** | **Sample Size** |
| --- | --- | --- | --- | --- | --- | --- | --- | --- | --- | --- | --- | --- |
| Marseille (2001) | CEA | Modelling | Public sector health payer | Not Specified | South Africa | # of HIV, syphilis, & gonorrhea cases averted (& Net savings) | Stand-alone | Outreach | Government | FSW | 50% | 1000 CSW |
| Panovska-Griffiths (2014) | CEA | Modelling | Not Specified | 4 years (2004-2007) | South India | Infection averted | Stand-alone | Mixed | NGO | FSW | 15.60 | 3200 FSW |
| Prinja (2011) | CUA | Modelling | Health system | 20 years | India | DALYs & HIV infect. averted | Integrated | Mixed | Government | FSW | 1.00% | Not Specified |
| Sweat (2006) | CUA | Modelling | Implementing NGOs | 1 year | Dominican Republic | DALY averted | Stand-alone | Outreach | NGO | FSW | 6% | 10,000 |
| Tromp (2013) | CUA | Modelling | Government | 20 years (2010-2030) | Indonesia (west java) | DALY& infection averted | Integrated | Outreach | Government | FSW | 11.6% | Not Specified |
| Vassal (2014) | CUA | Observational (Retrospective) | Programme | 7 years (2004-2011) | South India | DALY averted | Stand-alone | Outreach | NGO | FSW | 16.4% | 150,000 |
| Vickerman (2006a) | CEA | Modelling | Provider | Not specified exactly (3 years) | Benin | HIV infect. averted | Integrated | Mixed | Government | FSW | 39% | 3200 FSW |
| Vickerman (2006b) | CUA | Modelling | Provider | 1 year (June 2000-June 2001) | South Africa (Johannesburg) | DALYs & HIV infect. averted | Stand-alone | Clinic | Government | All | 50% | 1431 FSW |
| Wilson (2010) | CUA | Modelling | Health sector | Life time | Australia | Inf. Averted & QALY gained | Stand-alone | Clinic | Government | All | 0.10% | Not Specified |
| You (2006) | CEA | Modelling | Provider | Not Specified | Hong Kong | Case of gonorrhea and chlamydia averted (in FSWs and in clients) | Stand-alone | Outreach | Government | FSW | Not Specified | Not Specified |
