# Supplementary material for: Cost effectiveness of HIV and sexual reproductive health interventions targeting sex workers: a systematic review
Source: Cost Eff Resour Alloc. 2018 Dec 4;16:63. doi: 10.1186/s12962-018-0165-0 (PMC6278021; doi:10.1186/s12962-018-0165-0)
Supplement: Supplementary file 4 — Additional file 4. Quality of reporting cost-effectiveness results for the interventions using CHEERS [file 12962_2018_165_MOESM4_ESM.docx]

Additional file 4: Quality of reporting cost-effectiveness results of the interventions using CHEERS*

| Item | | Item No | Aldridge 2009 | Burgos 2010 | Borghi 2005 | Carrara 2005 | Dandona 2010 | Fung, 2007 | Hogan 2005 | Hutton, 2013 | Leelahavarong, 2001 | Marseille 2001 | Panovska-Griffiths 2014 | Prinja, 2011 | Sweat, 2006 | Tromp 2013 | Vassal 2014 | Wilson 2010 | Vickerman, 2006a | Vickerman, 2006b | You 2006 |
| --- | --- | --- | --- | --- | --- | --- | --- | --- | --- | --- | --- | --- | --- | --- | --- | --- | --- | --- | --- | --- | --- |
| Title and abstract | Title | 1 | Y | Y | Y | P | Y | Y | Y | Y | Y | Y | N | Y | Y | Y | Y | Y | Y | Y | Y |
|  | Abstract | 2 | P | Y | Y | Y | P | Y | P | P | Y | Y | P | Y | Y | P | Y | P | P | P | Y |
| Introduction | Background and objectives | 3a | Y | Y | N | Y | P | Y | Y | Y | Y | Y | Y | P | Y | Y | Y | Y | P | Y | Y |
|  |  | 3b | Y | Y | Y | Y | Y | Y | Y | Y | Y | Y | Y | P | Y | Y | Y | P | Y | Y | Y |
| Methods | Target population and subgroups | 4 | Y | P | Y | Y | P | P | N | P | Y | Y | N | Y | N | N | Y | N | Y | Y | Y |
|  | Setting and location | 5 | N | N | Y | Y | P | P | Y | Y | Y | Y | Y | Y | Y | Y | Y | Y | Y | Y | Y |
|  | Study perspective | 6 | N | Y | Y | Y | N | Y | N | N | Y | Y | N | Y | Y | Y | Y | P | P | Y | Y |
|  | Comparators | 7 | Y | Y | Y | Y | N | P | Y | Y | Y | Y | P | P | Y | Y | Y | P | P | P | Y |
|  | Time horizon | 8 | N | Y | Y | P | Y | Y | N | N | Y | N | P | P | Y | Y | Y | Y | N | Y | N |
|  | Discount rate | 9 | N | Y | NA | P | NA | Y | NA | N | Y | Y | N | Y | Y | N | Y | P | P | NA | N |

* SRH: Sexual and reproductive health; CHEERS: Consolidated Health Economic Evaluation Reporting Standards; NA: not applicable; N: not reported; Y: reported; P: partially reported

Additional file 4- continued

| Item | | Item No | Aldridge 2009 | Burgos 2010 | Borghi 2005 | Carrara 2005 | Dandona 2010 | Fung, 2007 | Hogan 2005 | Hutton, 2013 | Leelahavarong, 2001 | Marseille 2001 | Panovska-Griffiths 2014 | Prinja, 2011 | Sweat, 2006 | Tromp 2013 | Vassal 2014 | Wilson 2010 | Vickerman, 2006 | Vickerman, 2006 | You 2006 |
| --- | --- | --- | --- | --- | --- | --- | --- | --- | --- | --- | --- | --- | --- | --- | --- | --- | --- | --- | --- | --- | --- |
| Methods | Choice of health outcomes | 10 | P | P | P | Y | Y | Y | P | P | P | Y | P | Y | Y | Y | Y | P | P | Y | Y |
|  | Measurement of effectiveness | 11 a) | NA | NA | P | P | P | Y | NA | NA | NA | NA | Y | NA | Y | NA | Y | NA | N | N | NA |
|  |  | 11 b) | P | P | NA | NA | NA | NA | N | N | P | Y | NA | P | NA | P | NA | N | NA | NA | P |
|  | Measurement and valuation of preference based outcomes | 12 | NA | Y | NA | NA | NA | NA | NA | NA | P | NA | NA | NA | NA | NA | NA | N | NA | NA | NA |
|  | Estimating resources and costs | 13 a) | NA | NA | Y | Y | N | NA | NA | P | NA | NA | NA | NA | Y | NA | Y | NA | NA | NA | NA |
|  |  | 13 b | Y | Y | NA | NA | NA | Y | P | NA | Y | Y | P | Y | NA | Y | NA | Y | P | Y | N |
|  | Currency, price date, and conversion | 14 | N | Y | Y | Y | Y | Y | N | P | Y | N | N | Y | Y | P | Y | Y | P | Y | N |
|  | Choice of model | 15 | NA | Y | NA | NA | NA | Y | NA | NA | P | N | P | N | N | N | P | Y | Y | Y | Y |
|  | Assumptions | 16 | NA | Y | NA | NA | NA | Y | NA | NA | Y | Y | Y | N | Y | N | Y | Y | Y | Y | Y |
|  | Analytical methods | 17 | NA | N | N | NA | N | N | N | N | N | N | Y | N | NA | N | NA | N | NA | NA | N |

*NA: not applicable; N: not reported; Y: reported; P: partially reported

Additional file 4-continued

| Item | | Item No | Aldridge 2009 | Burgos 2010 | Borghi 2005 | Carrara 2005 | Dandona 2010 | Fung, 2007 | Hogan 2005 | Hutton, 2013 | Leelahavarong, 2001 | Marseille 2001 | Panovska-Griffiths 2014 | Prinja, 2011 | Sweat, 2006 | Tromp 2013 | Vassal 2014 | Wilson 2010 | Vickerman, 2006 | Vickerman, 2006 | You 2006 |
| --- | --- | --- | --- | --- | --- | --- | --- | --- | --- | --- | --- | --- | --- | --- | --- | --- | --- | --- | --- | --- | --- |
| Results | Study parameters | 18 | Y | Y | Y | P | Y | Y | N | Y | Y | Y | Y | Y | Y | N | Y | Y | Y | Y | P |
|  | Incremental costs and outcomes | 19 | Y | Y | Y | N | P | Y | Y | Y | Y | Y | P | Y | Y | N | Y | N | Y | Y | P |
|  | Characterising uncertainty | 20 a | NA | NA | Y | P | Y | NA | NA | NA | NA | NA | NA | NA | Y | NA | NA | NA | NA | NA | NA |
|  |  | 20 b | Y | Y | NA | NA | NA | Y | Y | P | Y | Y | Y | Y | NA | Y | Y | P | Y | Y | Y |
|  | Characterising heterogeneity | 21 | NA | NA | Y | N | Y | NA | P | N | N | Y | Y | NA | Y | N | Y | Y | NA | Y | N |
| Discussion | Study findings, limitations, generalisability, and current knowledge | 22 | P | Y | P | Y | Y | P | Y | Y | P | Y | Y | Y | Y | P | Y | Y | Y | P | P |
| Other | Source of funding | 23 | Y | Y | Y | Y | Y | Y | Y | Y | Y | Y | Y | Y | Y | Y | Y | Y | Y | Y | Y |
|  | Conflicts of interest | 24 | Y | N | N | Y | Y | Y | Y | N | Y | N | Y | Y | N | N | Y | Y | N | N | N |

* NA: not applicable; N: not reported; Y: reported; P: partially report
