# Supplementary material for: Cost effectiveness of HIV and sexual reproductive health interventions targeting sex workers: a systematic review
Source: Cost Eff Resour Alloc. 2018 Dec 4;16:63. doi: 10.1186/s12962-018-0165-0 (PMC6278021; doi:10.1186/s12962-018-0165-0)
Supplement: Supplementary file 5 — Additional file 5. Sensitive analysis results and drivers of cost-effectiveness. [file 12962_2018_165_MOESM5_ESM.docx]

Additional file 5: Sensitive Analysis Results & Drivers of Cost-effectiveness

| **Paper** | **Type of sensitivity analysis** | **Variables used in Sensitivity Analysis** | **Variables that had an effect on Cost-effectiveness results** |
| --- | --- | --- | --- |
| Aldridge  (2009) | Multi-way analysis (not clear) | Low, average & high intervention effectiveness  Random HIV prevalence rates | Rates of condom use  Treatment of STIs  Number of sexual partners  Age at first sex. |
| Borghi (2005) | One way & best and worst case scenario | Local salaries  STI Cure Rate under voucher scheme  Price of Condoms  Average cost of outpatient visit  Cost of laboratory tests | Increases in service provision in the absence of the voucher scheme  Prices of resource inputs in voucher scheme  Condom prices |
| Burgos (2010) | One, two, multi-way and Probabilistic | All input values | HIV Incidence |
| Carrara (2005) | One way | Discount rate  Staff time  Local vs. Expatriate salaries | Local vs. Expatriate Salaries |
| Dandona (2010) | Probabilistic | All input variables changed randomly through Monte Carlo Simulations. | NA |
| Fung (2007) | Two-way | Cost and impact to establish a range for cost effectiveness ratio. | Peer educator time needed/cost  STD cofactor effect per sex act  HIV transmission probability per sex act  Average duration of STDs |
| Hogan (2003) | Multi-way analysis (not clear) | Program Input costs  Behavioral impacts | Program input costs |
| Hutton (2013) | Two-way | Rates of new infections  Cost per sex worker educated  Use of condom increase | Not Reported |
| Leelahavarong (2001) | One-way, Probabilistic & Threshold Analysis | Vaccine efficacy  Duration of vaccine protection  Vaccine acceptance rate  Change in risk behavior.  All other model parameters (probabilistic) e.g. input costs | Risk behavior post-vaccination  Vaccine efficacy  Duration of protection  Input costs |
| Marseille (2001) | Univariate & Multivariate Sensitivity Analysis | Effectiveness of Female Condoms (FC)  HIV Prevalence among FSW & clients  Partners per FC user  HIV transmission risk per episode  Period of syphilis & gonorrhea infectivity  Syphilis and gonorrhea prevalence & transmission risk  Male & Female Condom use rates respectively  Cost per male & female condom respectively  Cost per treating HIV, syphilis & gonorrhea respectively  Cost of FC including promotion costs. | Number of partners per FC user  HIV Prevalence amongst FSW & clients  Effectiveness of the FC  Costs of FCs including promotion costs |
| Panovska-Griffiths (2014) | Done but not clearly specified | Intervention scale  Intervention intensity  Consistency of condom use  HIV infectivity | Intervention scale  Intervention intensity |

Additional file 5-continued

| **Primary Author (publication year)** | **Type of sensitivity analysis** | **Variables used in Sensitivity Analysis** | **Variables that had an effect on Cost-effectiveness results** |
| --- | --- | --- | --- |
| Prinja (2011) | Univariate & Probabilistic | Number of FSW, clients and general population  Condom use  HIV Prevalence  Risk of STD & HIV Transmission  Intervention parameters (unit cost of targeted interventions, effectiveness of STI treatment)  Discount rate (3% to 8%)  Input costs  STD cofactor effect | Risk of STD & HIV transmission  STD Cofactor effect  Client size per Sex Worker  Unit costs to implement intervention  FSW condom use |
| Sweat (2006) | One-way, two-way, multivariate and probabilistic | HIV Prevalence  STI Prevalence  Probability of HIV transmission  Sexual behavior  Cost per client  Discount rate  Number of sex partners  Condom use rates | Discount rate  HIV prevalence  DALYs saved per HIV infection averted  Contact rate between female sex workers and regular partners. |
| Tromp (2013) | One-way sensitivity analysis with +/-25% parameter change | Impact of Voluntary Counseling & Testing (VCT) on condom use  All epidemiological variables  All behavioral variables  Clinic capacity  Years of projection  AEM fitting variables | Impact of VCT on condom use  Population size of clients of female sex workers (FSWs)  HIV prevalence in the general population  Condom use between FSWs and clients. |
| Vassal (2014) | Not Specified | STI Incidence  Condom use  Time between SRH tests | Time between tests. |
| Vickerman (2006a) | Univariate | Cost of test  Time to take Point of Care (POC) test  Salary cost of staff taking POC  Time needed to train staff  Number of tests use to train staff  Discount rate | Cost of POC Test  Sensitivity of POC test |
| Vickerman (2006b) | Univariate | Specific model inputs  Staff time for start-up period  Drug costs  All other input parameters | Proportion of women who were FSW  STI transmission probability and cofactors  HIV transmission probability  Staff time for start-up period |
| Wilson (2010) | Univariate | Changes in STI incidence  Changes in condom use  Average time between tests. | Changes in STI incidence  Changes in condom use  Frequency of testing |
| You (2006) | One-way, two-way & Probabilistic sensitivity analyses | Chlamydia & Gonorrhea infectivity period  Health seeking behavior amongst FSW  Percent of condom use  Acceptance rate of outreach clinic  Cost of outreach service  Direct medical costs  All clinical variables | Number of clients per day  Changes in percent of condom use post-counseling.  Attendance to outreach clinic vs. usual medical care |
